# TRPV1 in chronic pruritus and pain: Soft modulation as a therapeutic strategy

**DOI:** 10.3389/fnmol.2022.930964

**Published:** 2022-09-02

**Authors:** Asia Fernández-Carvajal, Gregorio Fernández-Ballester, Antonio Ferrer-Montiel

**Affiliations:** Instituto de Investigación, Desarrollo e Innovación en Biotecnología Sanitaria de Elche (IDiBE), Universidad Miguel Hernández, Elche, Spain

**Keywords:** chronic pain, pruritus, therapeutic targets, TRP channels, soft drugs

## Abstract

Chronic pain and pruritus are highly disabling pathologies that still lack appropriate therapeutic intervention. At cellular level the transduction and transmission of pain and pruritogenic signals are closely intertwined, negatively modulating each other. The molecular and cellular pathways involved are multifactorial and complex, including peripheral and central components. Peripherally, pain and itch are produced by subpopulations of specialized nociceptors that recognize and transduce algesic and pruritogenic signals. Although still under intense investigation, cumulative evidence is pointing to the thermosensory channel TRPV1 as a hub for a large number of pro-algesic and itchy agents. TRPV1 appears metabolically coupled to most neural receptors that recognize algesic and pruritic molecules. Thus, targeting TRPV1 function appears as a valuable and reasonable therapeutic strategy. In support of this tenet, capsaicin, a desensitizing TRPV1 agonist, has been shown to exhibit clinically relevant analgesic, anti-inflammatory, and anti-pruritic activities. However, potent TRPV1 antagonists have been questioned due to an hyperthermic secondary effect that prevented their clinical development. Thus, softer strategies directed to modulate peripheral TRPV1 function appear warranted to alleviate chronic pain and itch. In this regard, soft, deactivatable TRPV1 antagonists for topical or local application appear as an innovative approach for improving the distressing painful and itchy symptoms of patients suffering chronic pain or pruritus. Here, we review the data on these compounds and propose that this strategy could be used to target other peripheral therapeutic targets.

## Introduction

Pain and itch (also known as pruritus) are nocifensive vital mechanisms that act as protecting alarm signals against tissue/organ damage that potentially threatens the integrity and survival of the organism. Itch and pain are distinct sensory modalities encoded by nociceptive subdivisions that interact at the level of the dorsal horn (Braz et al., 2014). Pain elicits a withdrawal response, while itch leads to scratching. Under physiological conditions, an antagonistic interaction exists between pain and pruritus such as scratch-induced pain often inhibits itch sensation. Conversely, pain inhibition with opioid analgesics elicits itch as a secondary effect (Ko, 2015). However, pain and itch also share many similarities, especially in chronic pathophysiological conditions that lead to the sensitization of nociceptive pathways.

Both pain and itch can be classified as either acute or chronic, depending on the duration of the symptoms (Grichnik and Ferrante, 1991). Acute pain and itch serve an important protective function as sentinel signals against potentially harmful external physical and/o chemical noxious stimuli such as insect and plant toxins, sharp objects, and noxious temperatures (Moore et al., 2018). The withdrawal response to pain avoids tissue damage, and the scratching activity favors removing the pruritogenic agent. On the other hand, chronic pain and itch are symptoms present in many disorders and, by themselves, may be considered as diseases rather than plain symptoms (Yosipovitch et al., 2007). Indeed, a common feature of chronic pain and itch is that the somatosensory system does not properly desensitize when the sensitizing noxious stimuli and/or the injury has healed. Thus, the system remains largely sensitized in the absence of a triggering event, becoming a disease by itself.

Chronic pain is a sensory condition that may be classified depending on the main cause as inflammatory, neuropathic, and psychogenic. Inflammatory pain is mediated by the presence of inflammatory/algesic agents that induce both peripheral and central sensitization of the somatosensory system leading to a persistent neural activity. Examples of inflammatory pain are arthritic pain, migraine, and inflammatory bowel disease (Guan et al., 2016). Neuropathic pain is caused by damage to the peripheral nervous system that also leads to a sensitization of nociceptors increasing their excitability. Conditions such as surgery, diabetes, chemotherapy, and herpes may lead to chronic neuropathic pain (Guan et al., 2016). Psychogenic pain, on the other hand, is a diffuse type of pain with a yet unclarified cause. Fibromyalgia has been classically classified as a diffuse type of pain, although the presence of a peripheral component has been proposed (Vecchio et al., 2020).

Chronic pruritus is a typical sensory symptom of skin disorders such as atopic dermatitis, psoriasis, notalgia paresthetica, prurigo nodularis, and urticaria. Likewise, pruritus is also present in systemic conditions, including hepatic cholestasis, diabetic neuropathy, kidney failure, and lymphomas. It is quite common that chronic pruritus manifests as episodic and spontaneous, with minimal or absent nociceptive symptoms interepisode.

Intense research in the past years has started to unveil the cellular and molecular mechanisms involved in the pathogenesis of chronic pain and pruritus, and to delineate the signaling pathways involved in the maintenance of nociceptor sensitization upon the triggering condition has resolved. Cumulative evidence signals to the transient receptor potential (TRP) channel superfamily as a central player in somatosensory signaling, particularly TRPV1 (Li and Wang, 2021). The TRP superfamily is composed of 28 members divided into six subfamilies, classified as canonical (TRPC), vanilloid (TRPV), ankyrin (TRPA), melastatin (TRPM), polycystin (TRPP), and mucolipin (TRPML) (Venkatachalam et al., 2014). This family of ion channel proteins function as non-selective cation-permeable channels, with Ca^2+^ permeability (Julius, 2013). In general, TRP channels function as molecular sensors of multiple physical and chemical stimuli, including changes in pH, chemical irritants including pungent peppers, wasabi, mustard, and menthol. They also respond to thermal, mechanical, osmotic, and actinic (radiation) cues.

Twenty years after its cloning, TRPV1 has become the first family member with a postulated and subsequently verified link to chronic pain and pruritus with increasing evidence that considers this receptor as a hub for algesic and pruritogenic agents (Li and Wang, 2021). This evidence is further supported by the analgesic, anti-inflammatory and anti-pruritogenic activities of capsaicin (Basith et al., 2016). Notwithstanding the role of TRPV1 in pain and itch signaling, the development of clinically useful receptor antagonists has been precluded due to their hyperthermic side effects, most likely due to a role of this channel in body temperature homeostasis (Yonghak et al., 2020). Thus, the development of TRPV1 modulators and, in general, molecules targeting other TRP channels, must consider acting preferentially on dysfunctional channels and avoid affecting the physiologically working population (Fernández-Carvajal et al., 2011). Alternatively, molecules that softly modulate channel activity may be a useful alternative to control peripheral channel activity. Here, we revise this important topic and describe the pre-clinical validation of soft TRPV1 antagonists to treat peripheral chronic pain and pruritus.

## TRPV1 structure and function

**FIGURE 1:**
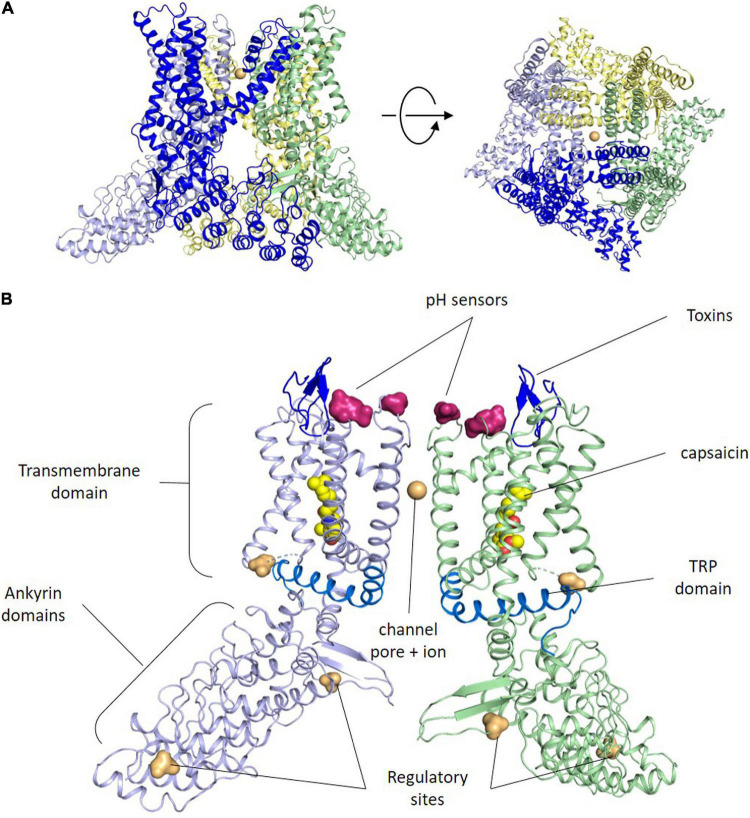
TRPV1 structure. **(A)** Side and upper views of tetrameric arrangement of the full human TRPV1 in the closed state. Each subunit of the TRPV1 channel is made up of six transmembrane domains. The pore loop is located between the S5 and S6 segments. Both the N and C termini are located Intracellularly. Sodium ions are shown as orange spheres. Contacts between monomers occur mainly in the membrane domain and at the C-terminus coiled–coil domain that lies around the central axis and surrounded by the N terminus. **(B)** Side view of the ribbon structural model of two opposite monomers of TRPV1 channel. The other two monomers are not shown for clarity. Amino acid residues specific for capsaicin and proton activation, as well as residues important for toxins binding, phosphorylation by protein kinases A and C (*PKA*, *PKC*) and Ca^2+^-calmodulin-dependent kinase II (*CaMKII*) kinases are highlighted at both intra-cytoplasmic regions.

The TRPV1 or capsaicin receptor was cloned by D. Julius in 1997 from a rat cDNA library (Caterina et al., 1997). Three years later the human ortholog was isolated exhibiting high similarity in sequence and function (Hayes et al., 2000). TRPV1 is an integral membrane protein composed of 4 subunits that usually assemble in homotetrameric ion channels (Figure 1A), although heterotetramers with other TRP channels have been reported (Cheng et al., 2012). TRPV1 channel assembly is highly stable and well preserved in detergent, which has facilitated its reconstitution into nanodiscs for 3D structure determination by electron cryomicroscopy (Liao et al., 2013; Gao et al., 2016).

Akin to other members of the TRP channel family, TRPV1 subunits exhibit an organization consisting of six-transmembrane helices (S1–S6) with a reentrant loop between S5 and S6 that forms the channel pore, the selectivity filter and an upper gate, while the C-terminal end of the S6 helix contains an internal gate (Cao et al., 2013; Figure 1B). The N- and C-termini of each subunit lie in the cytosol (Caterina et al., 1997), and are involved in modulating the channel sensitivity to activators and sensitizing agents, along with the oligomer stability and function (Jung et al., 2002; Schumacher and Eilers, 2010). For instance, the C-terminus has PKA, PKC, and Ca^2+^/Calmodulin-dependent phosphorylation sites that modulate receptor desensitization and tachyphylaxis. Furthermore, a highly conserved region in the C-terminus (referred to as the TRP domain) is implicated in subunit oligomerization (García-Sanz et al., 2007), and in the allosteric coupling between stimuli sensing and pore opening (Valente et al., 2008; Gregorio-Teruel et al., 2015). The N-terminus contains several ankyrin repeats that appear essential protein-protein interactions that stabilize the TRPV1 channelosome (Lishko et al., 2007).

Capsaicin, the pungent compound in chili peppers, is a vanilloid molecule that acts as a desensitizing agonist of TRPV1 (Tominaga and Tominaga, 2005). Other chemical substances activating TRPV1 are animal toxins (scorpions, snakes, spider, and jellyfish), divalent cations (Mg^2+^, Ba^2+^), endogenous substances (anandamide, leukotriene B, phospholipase C), natural molecules (piperine, gingerol, zingerone, camphor, and eugenol), and a long list of compounds still under investigation (extensively reviewed by Fischer et al., 2020). Furthermore, TRPV1 is also gated by noxious heat, with a temperature threshold of 42°C, extracellular acidic pH (<6,5) and depolarizing voltages (>80 mV). This diversity of activating mechanisms makes TRPV1 a polymodal ion channel responding to both physical and chemical stimuli. Notably, polymodality also allows to exploit the synergistic effect of chemical and physical stimuli. Hence, heat or voltage activation exhibit lower thresholds in the presence of capsaicin or extracellular acidic pH.

Upon activation by a ligand or physical stimuli, the channel opens through a dual gate mechanism involving changes in the selectivity filter as well as in the lower gate (Cao et al., 2013). The opening of the TRPV1 channel permeabilizes the cell membrane to ions in a non-selective manner, although with preference to Ca^2+^ ions that, in turn, activates intracellular signaling pathways that can lead to desensitization or sensitization depending on the environmental milieu and the machinery present in the cell.

TRPV1 desensitization appears due to channel closing by the prolonged presence of the activating stimuli, representing a mechanism to preserve cellular ionic homeostasis. This mechanism has been linked to a Ca^2+^/Calmodulin mechanism, involving C-terminus domain and mostly the inner gate of the channel (Rosenbaum et al., 2004). Additionally, the channel may suffer use-dependent desensitization or tachyphylaxis that involves the endocytotic removal of the protein from the neural membrane and its degradation by the proteasome (Sanz-Salvador et al., 2012). Channel desensitization and tachyphylaxis are the properties underlying the therapeutic use of capsaicin to treat chronic pain and itch (Arora et al., 2021).

**FIGURE 2:**
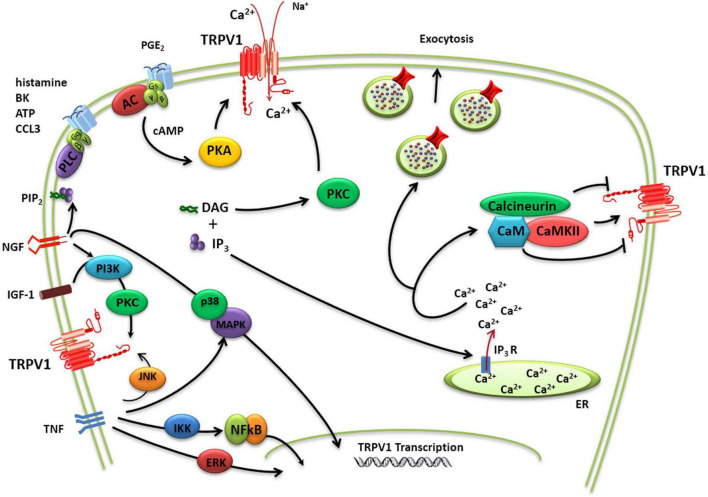
TRPV1 modulation. Schematic diagram of complex regulation of TRPV1 implicated in pain transduction. TRPV1 channels may not only act as ligand-gated ion channels but may also increase neuron excitability through the activation of intracellular signaling pathways. The activity of TRPV1 is controlled by a multitude of regulatory mechanisms that either causes sensitization or desensitization of the channel. TRPV1 sensitization appears mediated by direct phosphorylation of intracellular protein domains by PKC, PKA, and other kinases. A dynamic balance between phosphorylation and dephosphorylation of TRPV1 by Ca^2+^/calmodulin-dependent kinase II and calcineurin, respectively, appears to control the activation/desensitization state of the channel. TRPV1 activity may be additionally potentiated by Ca^2+^-induced exocytotic recruitment to the cell membrane of an internal pool of vesicular TRPV1.

TRPV1 activity may be sensitized by pro-inflammatory/algesic or pruritogenic agents (Shim et al., 2007; Figure 2). Channel sensitization is characterized by a notable increase of the channel activity partially due to a decrease in the threshold of temperature activation as a result of the chemical modification of the protein. TRPV1 sensitization appears mediated by direct phosphorylation of intracellular protein domains by PKC, PKA, and other kinases (Bhave et al., 2003; Varga et al., 2006; Figure 2). Protein phosphorylation may perturb the functional coupling mediated by the nearby TRP domain leading to lower thresholds for channel gating. Nerve growth factor (NGF) also contributes to TRPV1 sensitization increasing TRPV1 expression a process mediated by the p38/MAPK signaling pathway (Ji et al., 2002). In parallel, NGF activates phosphatidylinositol 3-kinase-protein kinase C (PI3K) and calmodulin-dependent protein kinase signaling cascades, increasing TRPV1 opening probability (Bonnington and McNaughton, 2003). Similar to NGF, insulin growthfactor-1 (IGF-1) also enhances TRPV1 membrane currents through PI3K and PKC pathways, increasing channel activity and receptor expression (Van Buren et al., 2005; Lilja et al., 2007; Figure 2). Furthermore, TNFα sensitizes TRPV1 channels *via* c-jun N-terminal kinase (JNK) pathway and p38/MAPK and PKC-dependent pathways and upregulates TRPV1 expression *via* ERK activation in cultured DRG neurons (Hensellek et al., 2007). In addition, p38 MAPK signaling has also been shown to increase TRPV1 channel expression following incubation with TNFα for periods of 1 h or greater (Constantin et al., 2008).

TRPV1 activity may be additionally potentiated by Ca^2+^-induced exocytotic recruitment to the cell membrane of an internal pool of vesicular TRPV1 (Morenilla-Palao et al., 2004; Van Buren et al., 2005; Camprubí-Robles et al., 2009; Figure 2). Notably, in peptidergic nociceptors TRPV1 appears to be transported to the peripheral terminals by large dense core vesicle that transport the calcitonin gene-related peptide (CGRP), a pro-inflammatory neuropeptide released in inflammatory conditions (Devesa et al., 2014). This co-transport of proalgesic peptides and TRPV1 appears as an elegant mechanism to rapidly sensitize the injured tissue while it is being repaired.

## TRPV1 distribution

TRPV1 expression and distribution has been reported in tissues and organs from human, rat, mouse, and other mammals (Krause et al., 2005). In rodents, the receptor was found in areas of the central nervous system (CNS) such as the hippocampus, cerebral cortex, cerebellum, central amygdala, thalamus, hypothalamus, cochlear nuclei, spinal nucleus of the trigeminal nerve, inferior olive, and the spinal cord (Mezey et al., 2000; Sawamura et al., 2017). In the peripheral nervous system, TRPV1 was detected in the trigeminal ganglion and in DRGs. TRPV1 was also reported in the mucosal epithelial cells, and other non-neural cells such as epidermal keratinocytes, infundibulum of hair follicles, T-cells, mast cells, macrophages, and leukocytes, and sweat gland cells (Bevan et al., 2014). In the cardiovascular system, expression of TRPV1 was found in cardiomyocytes, vascular endothelium (Gorbunov et al., 2019), and smooth muscle cells. Similarly, TRPV1 has been localized in a variety of human tissues including brain, spleen, kidney, small intestine, pancreas, testis, stomach, lung, liver, and urinary bladder (Storozhuk et al., 2019). Thus, TRPV1 is widely expressed suggesting a physiological role beyond thermosensation and detection of noxious heat stimuli that remains largely unresolved. This widespread tissue and organ distribution must be considered when developing TRPV1 antagonists as indiscriminate blockade of all channel populations may produce severe side effects that limit their therapeutic use.

## TRPV1 in the skin

The skin is the largest sensory organ that exhibits a very intense activity interacting with the environment. Apart from isolating tissues and organs for protection, it maintains a continuous chemical and physical surveyance of the environment that is essential for survival. For instance, in homeothermic organisms the skin continuously monitors the environmental temperature to maintain a constant body temperature; under heat conditions vasodilation and sweating is activated, while in cold environments vasoconstriction and piloerection are promoted. Notably, alteration of these regulatory mechanisms may lead to hyper or hypothermia (Schlader et al., 2009, 2011) that are lethal conditions.

**FIGURE 3:**
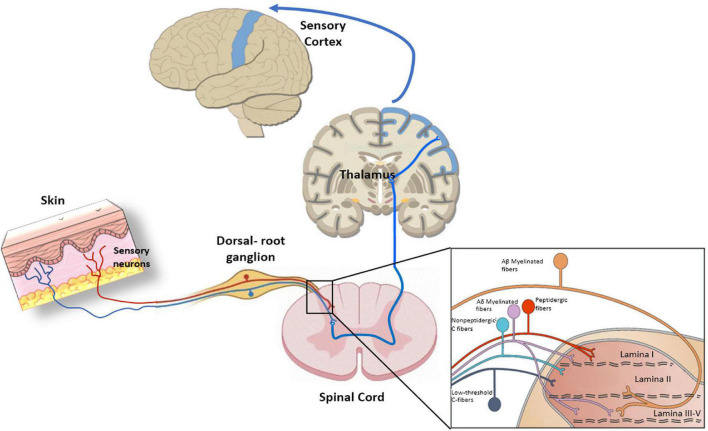
Peripheral nociception, Primary sensory neurons present a bifurcated axon with one branch innervating peripheral tissues and the other one reaching the central nervous system (CNS). The pathway starts with sensory neurons that synapse in the dorsal horn of the spinal cord. Next, neurons extend from the dorsal horn and decussate, or cross over to the other side of the spinal cord, before traveling up the spinal cord, through the brainstem, and to the thalamus. There, thalamic neurons integrate peripheral nociceptive information through projections to the somatosensory cortex, which processes the information. The insert indicates the organization of primary afferent fibers projecting to spinal cord dorsal horn. Nociceptive fibers terminate mainly in superficial dorsal horn (laminae I–II), while tactile afferents [Aβ and LT (low threshold) Aδ] project mainly to deep dorsal horn (laminae III–V). Nociceptive projection neurons are located in lamina I, while wide dynamic range neurons, activated by both tactile and nociceptive stimuli, are localized in lamina V.

The skin is largely innervated at the epidermis and dermis with diverse neural terminals that endow this organ with its remarkable sensitivity to detect environmental innocuous physical and chemical signals, to alert the presence of noxious stimuli that may harm tissues, and to trigger a proper nocifensive response (Bagood and Isseroff, 2021). Skin sensory nerves can be categorized as myelinated Aβ and Aδ-fibers, and unmyelinated C-fibers. Aβ are fast transmitting fibers mainly involved in the detection of mechanical stimuli, while Aδ-fibers have slower velocities and can also detect thermal signals (Roosterman et al., 2006). Unmyelinated C-fibers are polymodal slow conducting fibers primarily involved in the detection of noxious stimuli and warm sensation at the epidermis (Paricio-Montesinos et al., 2020). All peripheral fibers project into the lamina I and II of the spinal cord to connect with the ascending pathways (Koch et al., 2018; Figure 3).

Unmyelinated C-fibers are made of sensory neurons called nociceptors that can be subdivided as peptidergic or non-peptidergic. Peptidergic nociceptors are characterized by exhibiting large dense core vesicles loaded with neuropeptides CGRP and substance P (SP) that are released in response to noxious stimuli. Both nociceptor types are polymodal responding to physical and chemical stimuli and are sensitized by pro-algesic/inflammatory and pruritogenic agents. Nociceptor sensitization is translated into a notable increase in their electrical excitability and involves transcriptional, translational and functional changes that decrease their firing threshold leading to an augment in spontaneous and tonic firing (Villalba-Riquelme et al., 2022).

**FIGURE 4:**
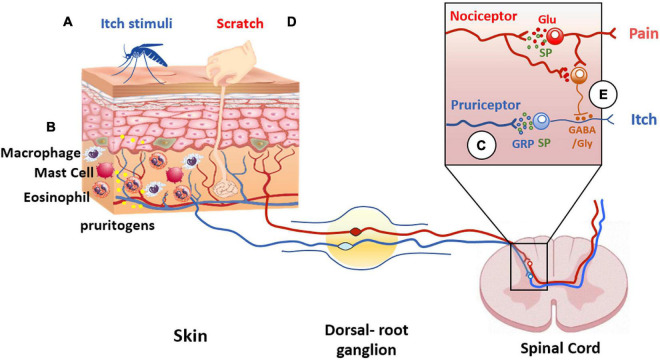
Itch and pain pathway interaction. Pruritogens **(A)** initiate itch sensation *via* pruriceptors (blue line) activation present in cutaneous sensory neurons. **(B)** In addition, other cell types are involved in itch signaling, such as keratinocytes, or innate immune cells that release pruritogens (yellow dots) such as histamine and IL-31, leading to the activation of pruriceptors and induction of peripheral sensitization. **(C)** The intraspinal terminals of pruriceptors (blue line in insert) release gastrin releasing peptide (GRP) (blue dots) and/or substance P (SP) (green dots) as neuropeptide transmitters to excite post-synaptic neurons expressing GRP and/or NK-1 receptors. **(D)** Scratching the skin presumably activates mechanically sensitive C-fiber polymodal nociceptors with primary afferent projections to the superficial dorsal horn of the spinal cord (red line). These afferents release glutamate to excite inhibitory interneurons in the superficial dorsal horn resulting in inhibition of the itch pathway **(E)**. The inhibitory interneurons use glycine and GABA as neurotransmitters to inhibit pruritogen-responsive neurons (orange dots).

Notable, C-fibers interact with a plethora of cells including mast cells, Langerhans cells, blood vessels, and keratinocytes (Hilliges et al., 1995; Figure 4). This interaction is largely uncharacterized but illustrates an intimate communication of nerve terminals with immune and cutaneous cells that shape the skin functionality (Vidal Yucha et al., 2019). Dysregulation of this communication due to tissue injury or reactivity of the immune system significantly contributes to the pathophysiology of peripheral disorders and their chronification. Indeed, pruritogenic factors released by dermal and immune cells sensitize peripheral terminals that, in turn, release pro-inflammatory peptides that stimulate dermal cells creating a feedback loop that enhances and prolongs the conditions that may lead to central sensitization (Mehta and Granstein, 2019). In this regard, TRPV1 is highly expressed by peripheral sensory nerves in the skin, as well as central nerve endings in the dorsal root ganglia (Bagood and Isseroff, 2021).

## TRPV1 in pain

Peripheral TRPV1 appears to act as a hub for pro-algesic, inflammatory, and pruritogenic agents that increase both its expression and function leading to enhanced neuronal excitability (Wu et al., 2013). Cumulative evidence supports that TRPV1 pivotally participates in pain evoked by noxious chemicals and heat and contributes to peripheral sensitization (Willis, 2009; Malek et al., 2015). Some agents of the inflammatory soup reduce the activation threshold of TRPV1 near the body temperature, which results in the firing of normally silent nociceptors. Complementarily, pro-algesic agents may also increase the surface expression of TRPV1 by mobilizing a subcellular reservoir near the plasma membrane that potentiate nociceptor activity (Camprubí-Robles et al., 2009). In chronic conditions, an increment in the receptor transcription and translation can also occur that contributes to prevent nociceptor desensitization upon resolution of the triggering sensitizing event (Xing et al., 2019; Bryk et al., 2020). Studies of TRPV1 function in models of neuropathic pain indicate that an increase in its function is coupled to an altered cell expression after peripheral nerve injury (Arribas-Blázquez et al., 2019; Villalba-Riquelme et al., 2022).

The implication of TRPV1 in peripheral pain transduction encompasses cutaneous and visceral pain as this thermoTRP channels also has a critical role in monitoring the proper functioning of our viscera (i.e., gut, bladder, pancreas, etc.). In both cases, downregulation of TRPV1 activity produces an anti-nociceptive action (Ponsati et al., 2012; Serafini et al., 2018; Huang et al., 2020).

The role of TRPV1 in non-neuronal cells in the skin, including immune cells, keratinocytes, melanocytes, adipocytes, and sebocytes is still under intense investigation and may be involved from cell proliferation/differentiation to the release of pro-inflammatory agents that in turn act on nociceptor peripheral ends sensitizing neuronally expressed TRPV1 channels that trigger pain signaling (Caterina and Pang, 2016; Mehta and Granstein, 2019). Thus, therapies targeting TRPV1 are useful reducing sensitized nociception as well as cutaneous disorders mediated by overactivity of the thermoTRP (Fernández-Carvajal et al., 2020).

## TRPV1 in itch transduction

Itch has been considered a submodality of pain, although currently both sensory modalities are distinguished as different (Jian et al., 2016). Pruritogens initiate itch sensation *via* activation of pruriceptors, a subclass of nociceptors, present in cutaneous sensory fibers (Xie and Hu, 2018). In addition, other cell types are involved in itch signaling, such as keratinocytes, or innate immune cells that release pruritogens that excite sensory terminals. Amines and cytokines 5-HT, SP, IL-31, and histamine, among others, are strong pruritogenic agents (Shim et al., 2007; Figure 4).

TPRV1 has been suggested to be involved in many chronic itch conditions like rosacea (Sulk et al., 2012), atopic dermatitis (Tang et al., 2022), and prurigo nodularis (Ständer and Schmelz, 2006). TRPV1 plays a crucial role in itch induced by histamine, the best known endogenous pruritogen. Histamine type 1 receptor (H1R) coexpresses with TRPV1 in pruritogenic nociceptors, and its signaling is coupled to TRPV1 activation, inducing membrane depolarization and activation of Ca^2+^-dependent intracellular cascades (Kim et al., 2004; Figure 2). Direct activation of TRPV1 by pruritogens (cyclic acid phosphatidic) also induces scratching reflex, that are antagonized by monounsaturated fatty acids and oleic acid (Morales-Lázaro et al., 2016). Other TRPV1-mediated itch signaling pathways include protease-activated receptor 2 (PAR2), vesicular glutamate transporters (VGLUTs), Toll-like receptor (TLR7) (Kim et al., 2011), and the TRPV1-binding protein Pirt (Kittaka and Tominaga, 2017). Complementarily, non-histaminergic pruritus such as that underlying in cholestasis has also been related to TRPV1 sensitization by pruritogens (Belghiti et al., 2013). Thus, TRPV1 appears to be a hub for pruritogenic signals contributing to histaminergic and non-histaminergic pruritus. This activity is partially shared with TRPA1 that has been involved in mediating some kinds of non-histaminergic pruritus (Tsagareli et al., 2020). Notably, TRPV1 and TRPA1 coexpress in most sensory terminals and they may also form heteromers which expands the involvement of TRPV1 as a pruritogenic signaling core (Fischer et al., 2014). Taken together, cumulative evidence signals to TRPV1 as a central therapeutic target for alleviating chronic pruritus.

## TRPV1 as a therapeutic target

The central role of TRPV1 receptor in pain and pruritus transduction has attracted the interest of pharmacology departments and pharmaceutical companies in the discovery and development of TRPV1 modulators (Wong and Gavva, 2009; Trevisani and Gatti, 2013; Jardín et al., 2017; Fernández-Carvajal et al., 2020). Genetic deletion of the receptor markedly reduced inflammatory thermal hyperalgesia and reduced itch (Moore et al., 2018). In the meantime, it was known in the clinic the analgesic, anti-inflammatory, and anti-pruritogenic activities of capsaicin, a desensitizing agonist of the receptor (Brederson et al., 2013). Topical application of capsaicin has been widely used because of its therapeutic properties that have been highly recognized by clinicians (Banerjee and McCormack, 2020). However, its burning sensation arising from its agonistic activity has been burdensome to patients limiting their adherence to the treatments. Thus, a notable public and private effort was launched to develop potent and selective oral TRPV1 antagonists and desensitizing agonists devoid of a burning sensation. This quest of TRPV1 antagonists resulted in the discovery of a large number of compounds that explored the chemical space leading to very potent and selective TRPV1 antagonists for treating a plethora of TRPV1 mediated disorders (Fernández-Carvajal et al., 2020). The first TRPV1 antagonist in clinical development was compound AMG517 (*N*-(4-((6-(4-(trifluoromethyl)phenyl)-4-pyrimidinyl)oxy)-2-benzothiazolyl)acetamide) developed by AMGEN (Doherty et al., 2007). This compound inhibited TRPV1 polymodal activation with low nanomolar potency and high receptor selectivity. Preclinical studies revealed a high oral availability and good pharmacological safety which prompted its clinical development. Unexpectedly, compound AMG517 produced significant hyperthermia in volunteers enrolled in a phase I clinical trial, which halted its clinical development (Gavva, 2008). A similar effect was observed for virtually all of the antagonists discovered by pharmaceutical companies that had to cancel or redirect the clinical development of their candidates (Kort and Kym, 2012).

Hyperthermia was not detected in preclinical studies nor in TRPV1 null mice, although posterior studies in rodents also detected a hyperthermic effect (Yonghak et al., 2020). Hyperthermia appears to arise from unselective blockade of physiologically and pathologically working TRPV1 receptors. Indeed, some studies have found that the TRPV1 receptor plays an important role, not yet fully elucidated, in the control of body temperature (Gavva et al., 2008). Therefore, indiscriminate TRPV1 inhibition in all tissues and modes of activation leads to dysregulation of the body’s thermal homeostasis. This finding has prompted the search for antagonists that preferentially block one activation mode (i.e., chemical or physical), without affecting the others (St Pierre et al., 2009), or that bind to drug sites that are exposed upon channel opening (i.e., a competitive antagonists) (Vidal-Mosquera et al., 2011). These strategies, however, have not resulted yet in oral antagonists in clinical development, emphasizing the challenge of developing safe systemic TRPV1 antagonists. Further progress in the pharmacology of TRPV1 is required for discovering TRPV1 antagonists for clinical use.

## TRPV1 topical modulation

Despite the apparent initial failure of TRPV1 drug discovery programs, clinical modulation of the TRPV1 receptor has been successfully accomplished using topical application of desensitizing agonists (Banerjee and McCormack, 2020). These compounds have exhibited excellent results alleviating chronic pain and pruritus. Currently, 8% capsaicin patches (Qutenza^®^) are used for the treatment of resistant neuropathic pain, peripheral neuropathies and cholestatic pruritus (Brederson et al., 2013). The pharmacological activity of topical capsaicin is very good, achieving remarkable symptom reduction. This is due to the ability of capsaicin to desensitize TRPV1 receptor and to promote receptor proteosomal degradation after prolonged exposure (Sanz-Salvador et al., 2012). However, because of its agonist activity, topical application of the vanilloid is initially accompanied by a very annoying burning effect that greatly distresses patients and prevents its use for chronic disorders. For instance, the Qutenza^®^ patches for treating neuropathic pain should be administered under local anesthesia to reduce the vanilloid burning sensation (Christensen et al., 2021).

Resiniferatoxin (RTX) is a natural compound from *Euphorbia resinifera*, which has a much higher affinity and desensitizing potency than capsaicin. RTX is a very potent desensitizing agonist that exhibits lower burning sensation than capsaicin. Local application of this molecule has been shown to attenuate arthritic pain (Bert et al., 2016). In 2022, Grünenthal and NovaQuest disclosed an agreement to advance the resiniferatoxin global Phase III program in osteoarthritis. In addition, a Phase I Study of the intrathecal administration of RTX for treating severe refractory pain associated with advanced cancer is currently ongoing.^1^

An alternative to topical/local TRPV1 receptor desensitizing agonists, is the development of receptor antagonists. Asivatrep (PAC14028, (2E)-*N*-((1R)-1-(3,5-difluoro-4-(methanesulfonamido)phenyl)ethyl)-3-(2-propyl-6-(trifluoromethyl)pyridin-3-yl)prop-2-enamide) is an antagonist that blocks TRPV1 with a potency of 6.2 nM that the Korean company Amore Pacific has progressed into clinical trial for mild to moderate atopic dermatitis.^2^ This compound showed good safety and efficacy results (Park et al., 2022). However, recent news suggest that the clinical development of this product has been discontinued.^3^

An additional concern of vanilloid derivatives is their poor dermal elimination that leads to their dermal accumulation in long term treatments. Preclinical studies have shown that radiating capsaicin with intense UV may produce free radical metabolites with carcinogenic activity (Surh and Lee, 1995). Since capsaicin exhibits a very slow dermal elimination kinetics and accumulates in the skin upon repetitive applications (Babbar et al., 2009), frequent and intense solar exposure may induce the formation of these carcinogenic derivatives (Bley et al., 2012). This concern also applies to vanilloid competitive antagonists applied topically that may also accumulate in the dermis where solar UV may produce carcinogenic metabolites. In this regard, potential carcinogenic activity due to UV radiation of Asivatrep was evaluated *in vitro* with promising results (Choi et al., 2020). Therefore, the development of desensitizing agonists or antagonists must consider skin accumulation as a safety endpoint for a better therapeutic index treating chronic pain and pruritus.

## TRPV1 soft modulators

A therapeutic alternative for a safe modulation of pathologically acting TRPV1 channels should consider: (i) moderate potency with balanced kinetics (k_on_/k_off_), particularly a moderate-to-fast k_off_, i.e., fast released from the receptor binding to avoid use dependent receptor blockage that may indiscriminately downregulate physiologically working receptors; (ii) low dermal accumulation to prevent the formation of UV-induced metabolites; (iii) absence of pungency for patient adherence to the treatment; and, (iv) a localized tissue action. Drug molecules that exert their biological activity at the site of action and are adequately metabolized outside, known as soft drugs, represent an excellent option (Buchwald, 2020).

Soft drugs are bioactive molecules endowed with a metabolically labile link to undergo an enzymatic controlled deactivation directed to eliminate the excess of bioactive molecules helping to achieve a desired therapeutic effect with lower side effects. This allows the drug to act primarily in the dysfunctional region restricting migration to neighboring tissues as it will be metabolized outside the target tissue. These drugs are usually administered topically or locally (e.g., eyes, skin, nasal mucosa, bone articulations, lungs, or gastrointestinal track) and are rapidly inactivated upon leaving the site of action, virtually eliminating their systemic exposure and thus increasing their safety (Aprile et al., 2019). Accordingly, considering the pharmacological and therapeutic properties of capsaicin, vanilloid-based soft drugs could contribute to the development of safer and more effective TRPV1 modulators.

**FIGURE 5:**
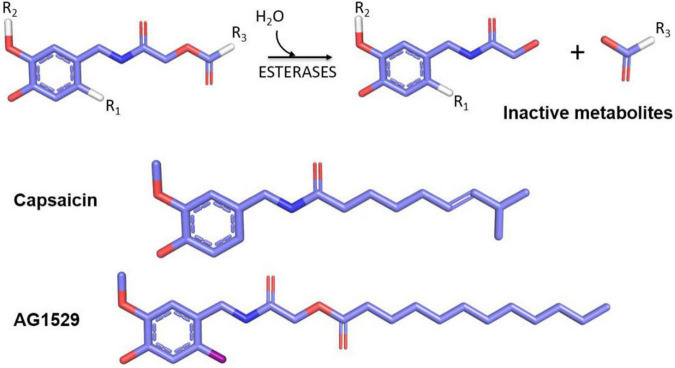
Soft drugs derived from capsaicin (upper part) have an ester bond sensitive to hydrolysis by skin esterases that produce an inert and easily disposable metabolite (vanillylacetamide) and a lipid that can be assimilated by cells. The inclusion of an iodine atom at the R1 position generates TRPV1 antagonists, and positions R2 and R3 may be used to increase the library chemical diversity. In the lower part, the comparative chemical structures between capsaicin and the selected AG1529 for further clinical development are shown.

In 2018, the soft drug principle was applied to capsaicin and a family of soft capsaicinoid analogs acting as desensitizing agonists and antagonists was designed, synthesized, and tested as a proof of concept of implementing the soft drug strategy to develop TRPV1 modulators with clinical utility. As a starting point, the capsaicin pharmacophore scaffold, composed of a vanilloid group (4-hydroxy-5-methoxy benzyl) linked to an octanoyl group *via* an amide bond was used (Serafini et al., 2018; Figure 5). The strategy consisted of replacing the amide bond with an ester bond linking the vanilloid group to the aliphatic region. The inclusion of the ester bond endowed the molecules with sensitivity to hydrolysis by esterases, releasing the vanilloid group that exhibits a high rate of dermal and renal elimination, and an aliphatic group formed by a fatty acid easily assimilated and metabolized by dermal cells. Chemical diversity was increased by modifying the fatty acid of the aliphatic region. This series of compounds included both desensitizing agonists and antagonists, with a shift in the activity provided by the insertion of an iodine atom at 6-position of the aromatic core (R1) (Figure 5).

Among these compound libraries, a family of soft TRPV1 antagonists was selected for further development. At variance with desensitizing agonists, receptor antagonists do not exhibit a burning sensation when applied topically or locally. The most potent TRPV1 antagonist, AG1529 (Figure 5), showed an IC_50_ value of 0.1 μM, and a significant specificity toward TRPV1, with a modest cross-reactivity with TRPA1 and TRPM8 channels, and no interaction with Nav and Kv channels (Nikolaeva-Koleva et al., 2021). This marginal cross-inhibition of thermoTRPs may have added pharmacological value for the treatment of multifactorial symptoms such as those present in chronic pruritus and pain.

The study of AG1529 activity in cultured rat primary sensory neurons revealed that this compound did not affect the firing of electrically evoked action potentials, but potently inhibited those elicited by capsaicin application and moderately those induced by extracellular acidification. It also showed modest inhibitory activity on action potentials evoked by TRPA1 activation. Overall, the functional properties of compound AG1529 made it a leading compound for further preclinical development. This capsaicin competitive antagonist exhibits a good *in vitro* pharmacological profile both in its potency and inhibitory efficacy, as well as in its preference for inhibiting the chemical activation modalities of the TRPV1 receptor, and a modest action on other thermoreceptors present in afferent terminals.

Preclinical studies showed that compound AG1529 markedly reduced neuronal excitability *in vitro* promoted by exposure of sensory neurons to histamine or to an inflammatory soup containing histamine. AG1529 decreased the activity of the TRPV1 receptor activity enhanced by the pruritogenic agent. Additionally, data on its metabolic stability confirmed that the compound was sensitive to dermal esterases, resulting in full hydrolysis, which limited its accumulation in the skin (Serafini et al., 2018).

Moreover, results obtained in animal models of histamine- or chloroquine-induced itching showed that topical application of AG1529 reduced scratching of the itchy region in a dose-dependent manner. Regarding its ability to reduce pain, preclinical studies were conducted in animal models of inflammatory pain induced by intramuscular administration of Freund’s adjuvant (CFA). These experiments revealed that compound AG1529 administered intraperitoneally or intravenously was able to eliminate the thermal hyperalgesia in the ipsilateral paw produced by CFA without affecting the temperature threshold of the contralateral paw. In addition, intravenous administration of 20 mg/kg did not raise the body temperature of the animals (i.e., did not produce hyperthermia), nor showed toxicity symptoms (Serafini et al., 2018). These results suggest a pharmacological effect on inflammatory pain and a good toxicity profile of TRPV1 soft antagonists.

Based on the above results, compound AG1529 has been selected as a candidate for clinical development for the treatment of psoriatic pruritus, a pathology that affects 3% of the population. In 2019, the spin-off AntalGenics, started the preclinical development of pharmacological safety and stability, as well as chemical characterization of the compound. Currently, preclinical studies are being concluded showing a good pharmacological safety and chemical stability profile, in order to apply for an IMPD (Investigational Medicinal Product Dossier) to the EMA (European Medicines Agency) to the start of clinical studies in 2023. Furthermore, less potent analogs have been developed as cosmeceuticals for skin care, particularly sensitive skin.

## Conclusion

Nociception is an important physiological process for detecting harmful signals that results in pain and itch perception. There is extensive experimental evidence involving TRPV1 ion channels, among other members of TRP family, as molecular sensors of chemical, thermal, and mechanical noxious stimuli to evoke pain and itch sensation.

Given that both sensations are evolutionarily refined protective mechanisms that favor survival, care should be exercised when developing inhibitory/modulatory compounds targeting specific pain/itch-TRPs to prevent inactivation of protective physiological mechanisms. This condition has limited the development of safe and effective TRPV1 modulators as the focus has been to obtain very potent and selective antagonists that block all functional receptors (i.e., knocking out strategy). Thus, medicinal approaches directed to design molecules that readily dissociate from TRPV1 channels (and in general, from the targeted neural receptor or channel), limiting use-dependent blockade of physiologically working receptors, and exhibiting a bias toward the pathologically altered TRPV1 population appear more attractive and define a safer disease-modifying approach. Hence, the soft drug approach for skin therapeutics that reduce pain and pruritus represent an elegant and safe strategy for locally alleviating these disabling symptoms. Therefore, soft drugs targeting cutaneous thermoTRPs may represent the next generation of therapeutic drugs for the treatment of disorders such as dermatitis, psoriasis, notalgia paresthetica, urticaria, prurigo nodularis, herpetic neuropathy, and chemotherapy-induced peripheral neuropathy. The soft drug approach may be extended to other neural and non-neural receptors. Note that the action of this approach maybe finely tuned by modulating the kinetics of esterase hydrolysis, extending from an epidermal to a dermal or deeper sites of action.

## Author contributions

All authors wrote the manuscript, contributed to the article, and approved the submitted version.

## Footnotes

1
https://clinicaltrials.gov/ct2/show/NCT00804154

2
https://clinicaltrials.gov/ct2/show/NCT02757729

3
https://adisinsight.springer.com/drugs/800048615
